# Supplementary material for: Alr Gene in Brucella suis S2: Its Role in Lipopolysaccharide Biosynthesis and Bacterial Virulence in RAW264.7
Source: Int J Mol Sci. 2023 Jun 28;24(13):10744. doi: 10.3390/ijms241310744 (PMC10341839; doi:10.3390/ijms241310744)
Supplement: Supplementary file 1 [file ijms-24-10744-s001.zip › ijms-2467920-supplementary.pdf]

TABLE S1 | Primers used in this study for LPS gene.

Gene ID	Primers	Sequences (5'- 3')
BMEI1427	RT-*wbkD*-F	TATTACGGACGGCTGGCTGA
	RT-*wbkD*-R	CACTACCGAACCGTTGGAGC
BMEI1426	RT-*wbkF*-F	CAGTCCACCGACGACCCACA
	RT-*wbkF*-R	CTTGCGCTAATTCCCGCTATCC
BMEI1418	RT-*wbkC*-F	CGATAACGCCACCGAAAGGA
	RT-*wbkC*-R	GTTGAAGAAACCGATACAGCC
BMEI1417	RT-*wbkB*-F	AGTTTGCGCTGCAATTCGTA
	RT-*wbkB*-R	GGCAATCAAACGATCCCGGT
BMEI1416	RT-*wzt*-F	CGAGATCCGCGAACTCGATG
	RT-*wzt*-R	GCCACGTTTCATGCGAGAGA
BMEI1415	RT-*wzm*-F	GCGCCCACGTAAATCAGACA
	RT-*wzm*-R	TGTCTGGAAGGTACGCCACT
BMEI1414	RT-*per*-F	GATGTAGTGCCACCGTTCCG
	RT-*per*-R	GGATCTCGTCGAGGGGTGAA
BMEI1404	RT-*wbkA*-F	GGCTTGCAGATCGCTTCCTT
	RT-*wbkA*-R	AAGTGCGAGACACCCGGATA
BMEI1396	RT-*manCO Ag*-F	GCAACGGTCCGTCAATTTGC
	RT-*manCO Ag*-R	CATTCCAGCCAGTGTGTCCC
BMEI1395	RT-*manBO Ag*-F	CCGAATCGCTATTCCTCGCC
	RT-*manBO Ag*-R	GCCATGGGCTCCAGAATGAC

Note: The gene IDs in the table are derived from *Brucella melitensis bv. 1 str.* 16M, as the specific gene IDs for *Brucella suis* S2 were not available. However, all the genes in this table have been subjected to homology alignment, and corresponding sequences for these genes are present in *Brucella suis* S2.

To validate the stable inheritance of the deletion strain, we designed upstream primers on the upstream homologous arm of alr and downstream primers on the KANAR fragment, as indicated in Table 1 of the original paper. The PCR product was 2229 bp in length. Furthermore, we performed continuous testing for 20 generations, demonstrating the consistent heritability of the deletion strain.

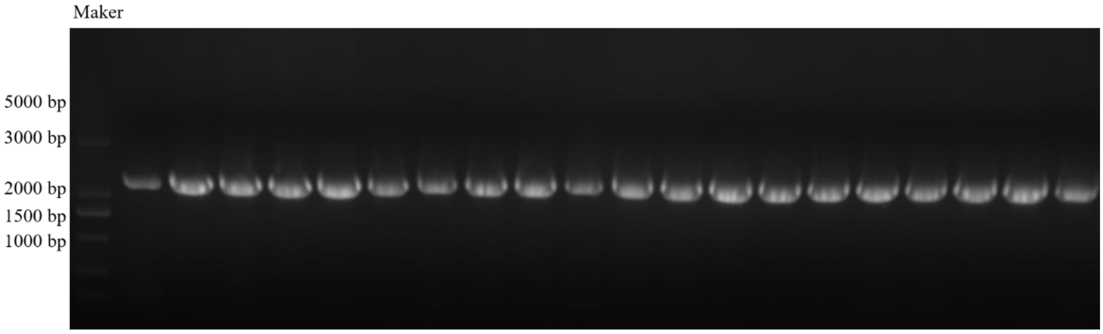

Figure S1: Stable Inheritance Verification of the Deletion Strain Marker: 5000 bp and Validation of the Deletion Strain for 20 Generations.
